# Five rules for resistance management in the antibiotic apocalypse, a road map for integrated microbial management

**DOI:** 10.1111/eva.12808

**Published:** 2019-05-14

**Authors:** Ben Raymond

**Affiliations:** ^1^ University of Exeter Penryn UK

**Keywords:** AMR, antibiotic resistance, antimicrobial stewardship, evolution of resistance, monotherapy, mutation supply, preventative action, resistance management

## Abstract

Resistance to new antimicrobials can become widespread within 2–3 years. Resistance problems are particularly acute for bacteria that can experience selection as both harmless commensals and pathogenic hospital‐acquired infections. New drugs, although welcome, cannot tackle the antimicrobial resistance crisis alone: new drugs must be partnered with more sustainable patterns of use. However, the broader experience of resistance management in other disciplines, and the assumptions on which resistance rests, is not widely appreciated in clinical and microbiological disciplines. Improved awareness of the field of resistance management could improve clinical outcomes and help shape novel solutions. Here, the aim is to develop a pragmatic approach to developing a sustainable integrated means of using antimicrobials, based on an interdisciplinary synthesis of best practice, recent theory and recent clinical data. This synthesis emphasizes the importance of pre‐emptive action and the value of reducing the supply of genetic novelty to bacteria under selection. The weight of resistance management experience also cautions against strategies that over‐rely on the fitness costs of resistance or low doses. The potential (and pitfalls) of shorter courses, antibiotic combinations and antibiotic mixing or cycling are discussed in depth. Importantly, some of variability in the success of clinical trials of mixing approaches can be explained by the number and diversity of drugs in a trial, as well as whether trials encompass single wards or the wider transmission network that is a hospital. Consideration of the importance of data, and of the initially low frequency of resistance, leads to a number of additional recommendations. Overall, reduction in selection pressure, interference with the transmission of problematic genotypes and multidrug approaches (combinations, mixing or cycling) are all likely to be required for sustainability and the protection of forthcoming drugs.

## 1 INTRODUCTION

Awareness of the current crisis in antimicrobial resistance (AMR) is widespread (Andersson & Hughes, 2014; CDC, 2013; Unemo & Nicholas, 2012). What is not yet clear is what we are going to do about it. Governments support reducing unnecessary prescriptions, a “rational use” philosophy; biochemists favour stimulating drug discovery while microbiologists and evolutionary biologists often argue for distinct stewardship approaches (CDC, 2013; Day & Read, 2016; Department of Health, 2013; Norrby et al., 2009; Walsh & Toleman, 2011; Worthington & Melander, 2013). Rationalizing usage is an important first step, but even given new drugs, how we will deploy them? No drug yet discovered is evolution proof (Bell & MacLean, 2018), while the typical practice of using single drugs at once, in unprotected “monotherapies,” is unsustainable. This “business as usual” approach of rolling out new drugs as older chemistries fail can be disastrous, as exemplified by the history of resistance in gonorrhoea and the emergence of untreatable infections (Unemo & Nicholas, 2012).

What is needed is a new philosophy in which usage is tied to a long‐term commitment to sustainability. Agriculture passed through a major crisis in resistance in late 1970s and 1980s, leading to the near collapse of the cotton industry in several countries (Kranthi & Russell, 2009). What emerged was the philosophy of integrated pest management (IPM), which emphasizes minimizing pesticide use and the diversification of management approaches. While IPM has not been universally applied, there has been increased commitment to reduce reliance on single modes of action, to reduce unnecessary selection pressure in the environment and to reduce applications, sometimes by a factor of 10 (Forrester, Cahill, Bird, & Layland, 1993). This change in philosophy, combined with several new modes of action, has been vital for 21‐century agriculture. We need a similar interdisciplinary effort for antimicrobials.

Integrated and multi‐tactic approaches to reducing carriage and transmission of multi‐resistant Gram‐positive bacteria in hospitals have already proven their worth (Derde et al., 2014; Huang et al., 2016) and can be seen as successful IPM of microbes. Based on the correlation between antibiotic usage and resistance (Costelloe, Metcalfe, Lovering, Mant, & Hay, 2010; Goossens, Ferech, Vander Stichele, & Elseviers, 2005), reduced prescribing should significantly lower resistance. Unfortunately, for some drugs (e.g., third‐generation cephalosporins) modest reductions in usage may not have noticeable effects (PHE, 2016). In farmed animals, resistance frequency declines with log_e_ antibiotic usage, so a fourfold reduction in usage only halves the prevalence of AMR genes (Munk et al., 2018). Moreover, historical withdrawals of antibiotics have had patchy impacts on the prevalence of resistance (Lipsitch, 2001). It is therefore likely that resistance management (RM) interventions beyond reduced prescribing will be required to tackle the crisis in antibiotic resistance.

This synthesis focuses on the problem of antibiotic resistance in bacteria, although the sources used to illustrate good and bad RM practice are varied. At the outset is important to emphasize where the key challenges lie. Although resistance can evolve by spontaneous mutation or horizontal gene transfer, resistance may be spread to a greater or lesser degree by transmission. When transmission is relatively unimportant and evolution is primarily spontaneous, the resistance management solutions are well known: combination therapy, that is, the use of multiple drugs simultaneously, is the most effective strategy (Monedero & Caminero, 2010; REX‐Consortium, 2013; Vandamme & Camacho, 2011), although the drawbacks of this approach are explored in more detail below. However, when transmission plays a larger role, solutions are not so clear‐cut (Bell & MacLean, 2018; Day, Huijben, & Read, 2015). There are added complications when bacteria can persist as harmless commensals in the gut (*Escherichia coli*, *Enterococcus* spp. *Klebsiellla* spp.) or in the nasopharynx (*Staphylococcus aureus*), as well as being able to cause life‐threatening hospital‐acquired infections. Commensals experience selection for resistance under a wide range of conditions, including treatment for other infections (Box 1). Commensals, particularly Enterobacteriaceae, make‐up the majority of the really problematic species in terms of emerging multidrug resistance (MDR; Livermore et al., 2011). This status means that resistance management interventions, especially in hospitals, should consider the forms of RM that are suited to commensals in addition to strict pathogens (Box 1 ; Lipsitch & Samore, 2002).

Box 1 **The contrasting dynamics of in vivo selection for infections with pathogens and for commensal bacteria that are not the main target of therapy**

1 For pathogens, antibiotic therapy has two possible outcomes, clearance or failure. Selection for resistance occurs if infected patients can transmit resistant microbes before clearance (or death). This means that early and effective treatment minimizes the spread of resistance, while treating infections with antibiotics for which there is pre‐existing resistance is the worst option (Beardmore, Pena‐Miller, Gori, & Iredell, 2017). Conversely, selection on the commensal microbiome typically accompanies the use of broad‐spectrum antimicrobials, since clearance is often not the aim of therapy. The simple dynamic figure suggests the main avenues for resistance management for commensals: minimize acquisition of resistance in preselection community via reduced transmission/reduced prescribing/heterogeneity of antibiotic use; reduce dosing period and duration of selection; or increase the rate of decline of resistant bacteria after selection, by increasing fitness costs of resistance or displacing resistant microbes with faecal transplants, for instance.

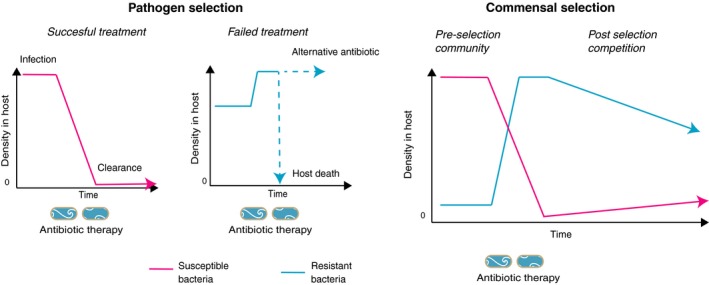

The aim of this article was to take a pragmatic approach to illustrate how resistance management principles could be more effectively applied to antibiotic‐resistant bacteria. If RM is badly applied, and leads to poor outcomes, then the reputation of RM approaches will suffer, making it likely that key decision‐makers will avoid powerful long‐term solutions to resistance, in favour of short‐term fixes. Here, an interdisciplinary perspective is used to illustrate common and well‐founded solutions to the evolution of resistance, such as the importance of pre‐emptive action and the value of heterogeneous selection and combinations (Bock & Lengauer, 2012; Boni, White, & Baird, 2016; Hughes & Andersson, 2015; Peck, 2001; Rex‐Consortium, 2013). Recent high‐profile or biotechnological approaches to resistance will also be critically evaluated in the light of the broader RM field and of key biological details. For convenience, key aspects of diverse RM interventions have been broken down into five rules.

## 2 RULE 1. PREVENTION IS BETTER THAN CURE

**Figure 1:**
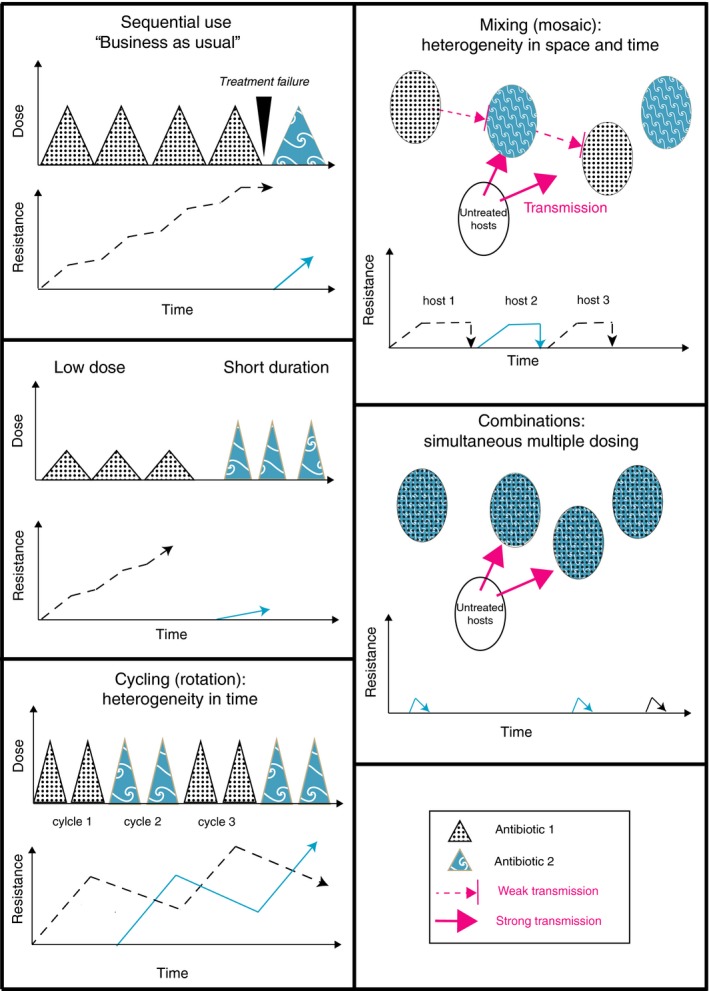
The major strategies employed in resistance management for microbes and other organisms with the predicted potential impact on the distribution of resistance. Where different terminology is in use in different disciplines, both terms have been provided (cycling = rotations; mixing = mosaics). Note that mixing and combination approaches work better if microbes are cleared from treated patients and if transmission occurs predominantly from untreated individuals. This can readily break down for commensal bacteria that are not the main target of therapy, but which can cause nosocomial infections in different individuals

**Figure 2:**
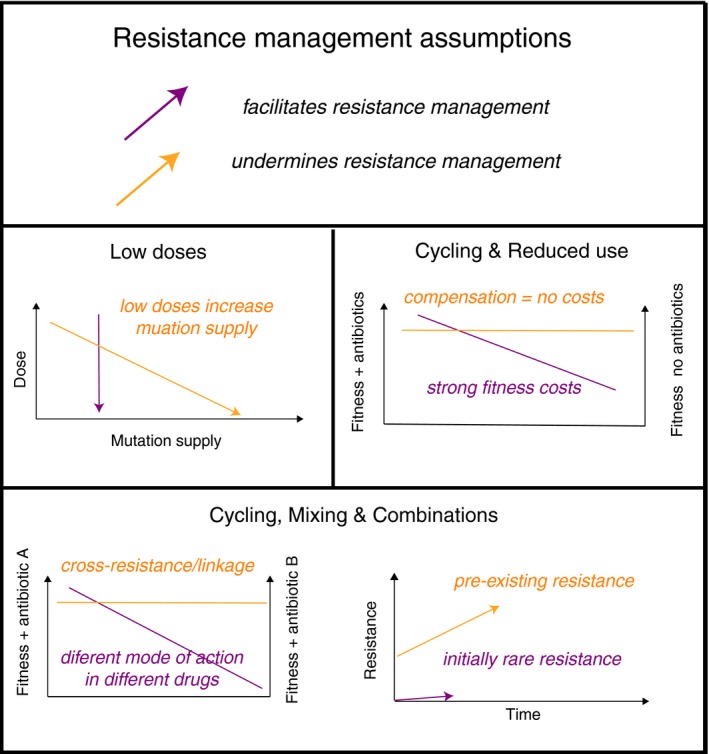
The critical assumptions of the major strategies of resistance management. Here, compensation refers to the process whereby additional mutations reduce the fitness cost associated with the acquisition of new mutations, or the costs associated with plasmids carrying resistance genes. All multidrug approaches rely on low initial frequencies and lack of cross‐resistance between drugs, where a single resistance trait give protection against multiple drugs. For combinations, ideally there must also be no synergistic interactions between drugs, and similar persistence in the body, see text for details

It is easy to misunderstand the limitations of resistance management interventions. A fundamental RM principle is that many more options are possible, while frequencies of resistance are still low (Boni et al., 2016). For example, a recent clinical trial evaluated two approaches to slowing the evolution of resistance in intensive care: the use of antibiotic “cycling” and “mixing” (van Duijn et al., 2018). In cycling, a particular class of antibiotic is used preferentially for a period of time followed by a different class, and so forth (Figure 1). In mixing, multiple distinct antibiotic regimes are prescribed in different patients, to create a spatial mosaic of antibiotic use. While it can be hard to make these approaches fully distinct in the clinic, they rely on different assumptions. Mixing aims at increasing the heterogeneity of selection pressure so that microbes resistant to one antibiotic cannot readily spread from patient to patient (Figure 1; Bonhoeffer, Lipsitch, & Levin, 1997). Cycling predominantly relies on resistance imposing a fitness cost in the absence of antibiotic selection and therefore  assumes resistance will decline when antibiotic use is suspended (Rex‐Consortium, 2013; Figures 1 and 2).

**Figure 3:**
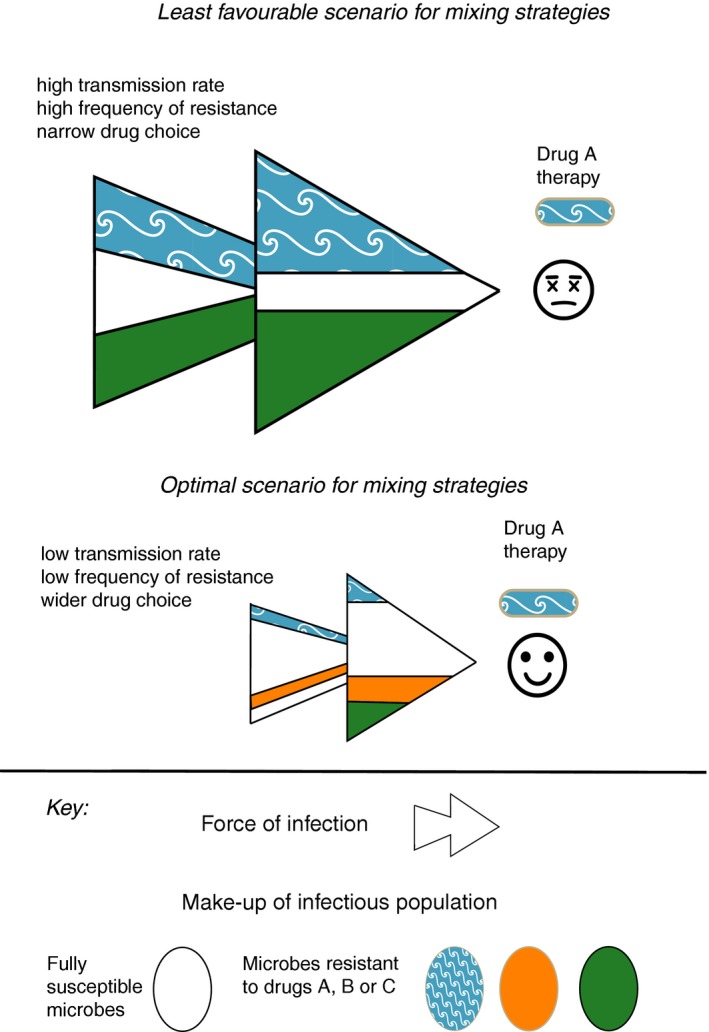
Transmission rates of bacteria will interact with resistance management strategies. Antibiotic mixing strategies rely on reducing the efficacy of transmission of resistant bacteria to new hosts, so that bacteria resistant to drug A are less likely to find themselves in a host being treated with drug A. Mixing strategies are unlikely to work under a high force of infection from a resistant microbial population, since patients may acquire multiple resistant microbes before antibiotic therapy begins (see Box). Broadening the transmission network to include more susceptible microbes should favour mixing, as would increasing the number of drugs in a mixing strategy

This latest trial is not alone in finding little evidence to support cycling and mixing in intensive care (Martinez et al., 2006; Sandiumenge, 2006; van Duijn et al., 2018). Nevertheless, a number of evolutionary factors may have opposed success. First, theory indicates that mixing is only beneficial when initial resistance frequencies are low (Bonhoeffer et al., 1997; Figure 2); with 28% of patients carrying antibiotic‐resistant Gram‐negative bacteria at the outset, this assumption is broken (van Duijn et al., 2018). High frequencies of resistance in many patients will generate a substantial force of infection, providing selectable diversity in untreated individuals (Box 1, Figure 3), especially if they can be colonized asymptomatically by MDR commensals. Second, multidrug RM should use chemistries with independent modes of action. Mixing/cycling three regimens that all use β‐lactams, albeit of different sub‐classes, can be problematic because some β‐lactamases (e.g., AmpCs; carbepenemases) can provide cross‐resistance to multiple drugs, and will be under selection in different regimens. Resistance to newer β‐lactam therapies such as piperacillin/tazobactam combinations is also often based on mutations in older β‐lactamases such as TEM‐1 (Lee, Oh, Choi, & Lee, 2013) so that the high prevalence of narrower spectrum β‐lactamases will facilitate the evolution of resistance to newer treatments. Increasing the number of antibiotic regimens in a mixing strategy should also be beneficial (Figure 3), although this has not been modelled explicitly. Successful trials of mixing strategies have employed six rather than three regimens and deployed structurally distinct carbapenems that can only be overcome by different resistance genes (Takesue et al., 2010). Nevertheless, since reversing resistance is particularly difficult, deployment of preventative strategies may be considered a success if they can stabilize levels of resistance (van Duijn et al., 2018).

A core aim of mixing strategies is the reduction in transmission of bacteria resistant to antibiotic A to new patients being treated with antibiotic A. It follows that deployment of mixing should consider the transmission networks of bacterial targets. Deploying mixing within a single ward is likely to be less powerful since transmission networks are likely to extend to the whole hospital: clinical trials of a standardized mixing regime were much more effective when deployed at a hospital level, rather than on a single ward (Takesue et al., 2010, 2006). Comparatively isolated wards such as intensive care units may also be difficult RM targets if they have relatively closed transmission networks, in other words if most transmission is from healthcare workers and patients within that ward. If a large part of this transmission is from patients with a high resistance burden, then this will make RM even more challenging. Transmission networks that include susceptible bacteria from unexposed individuals should be more amenable to mixing RM (Figure 3). More open transmission networks are known to reduce the residence time of commensal, resistant nosocomial specialists (Birgy et al., 2016), while being re‐infected with your own pre‐antibiotic microbiota is one means of increasing transmission from susceptible bacteria (Suez et al., 2018). This reasoning largely applies to commensals, as higher pathogen transmission and migration can increase infection rates and mutation supply for strict pathogens (Perron, Gonzalez, & Buckling, 2007).

## 3 RULE 2. FITNESS COSTS ARE UNRELIABLE SERVANTS

Fitness costs of resistance are undoubtedly important and can shape which mutations prevail in vivo (Linkevicius, Anderssen, Sandegren, & Andersson, 2016). Cycling RM and rotations, in general, rely on substantial fitness costs periodically driving down the frequency of resistance (Figure 1; Forrester et al., 1993). If fitness costs can be magnified, then these approaches should be more powerful. One means of magnifying costs is to exploit negative cross‐resistance, whereby resistance to one drug creates increases susceptibility to a second drug. Negative cross‐resistance associated with resistance mutations occurs in *S. aureus* and *E. coli* and can be exploited in vitro (Imamovic & Sommer, 2013; Kim, Lieberman, & Kishony, 2014)*.* It would be valuable to explore whether similar interactions exist in vivo for clinically important mutations. Resistance mutations in *Pseudomonas aeruginosa,* mutations in penicillin binding proteins in *Streptococcus* spp. and mutations conferring resistance to fluoroquinolones would be interesting avenues of study.

Negative cross‐resistance is also unlikely to be a feature of plasmid‐encoded resistance. Spontaneous resistance mutations often alter the shape of important proteins or RNA molecules, while plasmid‐encoded genes have less drastic impacts on particular biochemical functions, and so are less likely to create vulnerabilities to other drugs (Vogwill & Maclean, 2015), Moreover, massively increasing the fitness costs of particular resistance modes may simply alter the spectrum of prevailing resistance mutations, so that high‐cost alleles are replaced by lower cost alleles or by plasmid‐encoded traits (Linkevicius et al., 2016; Su et al., 2014). In insect pest management, successful exploitation of negative cross‐resistance is extremely rare (Pittendrigh et al., 2014) with a single example in the World Health Organization programme that managed the blackfly vector of onchocerciasis (river blindness) with three pesticide classes (Kurtak, Meyer, Orcran, & Tele, 1987).

Early experience of cycling drugs within patients was not an effective strategy for TB, as resistance did not decline rapidly when antibiotics were withdrawn (Fox, Ellard, & Mitchison, 1999). Changes in prescribing practice can lead to a rapid decrease in resistance via fitness costs (Whittles, White, & Didelot, 2017). However, when multiple resistance genes are co‐located on plasmids, withdrawl of one antibiotic may not lead to a decline in resistance, as selection from one drug can maintain resistance to many (Lipsitch & Samore, 2002). Plasmids themselves can be readily maintained in bacterial populations by conjugation. This means that withdrawal of a drug can lead to resistance declining very slowly, so that resistance persists at a level that enables a rapid response to selection when drug exposure resumes. It is also significant that the fitness costs associated with mutations or resistance plasmids are not necessarily stable. Ongoing selection commonly produces compensatory mutations that reduce these costs (de Vos et al., 2013; McNally et al., 2016). Since high prevalence of resistance implies multiple cycles of selection, this Rule may interact with Rule 1; it is better to act when resistance is rare and fitness costs are high. (see also Box 2).

## 4 RULE 3. LIMIT SUPPLY OF MUTATIONS AND GENETIC NOVELTY

Mutation supply is the product of mutation rate and bacterial population size. However, in order for mutations to be effective in overcoming resistance, they must confer a phenotype that can overcome the prevailing concentration of drug or drugs. Aside from mixing and cycling, the other key multidrug RM strategy is combination therapy, the simultaneous use of more than one drug in an individual (Figure 1). Combinations work because the simultaneous occurrence of multiple resistance mutations in a single microbe is very unlikely, that is, combinations reduce the supply of effective mutations. For instance, if mutations conferring resistance to rifampicin occur in 1 in 10^6^ bacterial cells, and to a second drug 1 in 10^8^ cells, then provided drugs have independent modes of action, and cells with mutations conferring resistance to both drugs occur a rate equivalent to the product of these frequencies, that is, 1 in 10^14^ cells.

Although these are simplistic calculations, combination therapy has been particularly effective for preventing resistance in microbes where spontaneous mutations dominate the evolution of resistance, notably in the treatment of human immunodeficiency virus (HIV) and tuberculosis (TB; Monedero & Caminero, 2010; Vandamme & Camacho, 2011). Combination therapy is also common in cancer treatment, where spontaneous evolution of drug resistance in human cells is extremely important (Bock & Lengauer, 2012). Combinations are also able to preserve the efficacy of resistance‐prone drugs such as rifampicin in the treatment of *P. aeruginosa* (Goss & Muhlebach, 2011). Combinations have been widely prescribed for clinical rather than RM reasons, for example, for severe life‐threatening infections. Unfortunately, the use of aminoglycosides in combinations can come with side‐effects (Paul, Lador, Grozinsky‐Glasberg, & Leibovici, 2014; Tamma, Cosgrove, & Maragakis, 2012), while combinations with fluoroquinolones increase risks of *Clostridium difficile* infections (Tamma et al., 2012). Nevertheless, it is not clear whether there are additional risks of using these drug classes in combinations versus single drug treatments (Tamma et al., 2012). Combinations are now recommended as part of resistance management of *Neisseria gonorrhoeae* (Bignell & Unemo, 2013). Drug combinations targeting *P. aeruginosa* and *S. aureus* have also yielded encouraging results (McCaughey, Diamond, Elborn, McKevitt, & Tunney, 2013). For TB, HIV and management of cystic fibrosis effective RM are absolutely fundamental for patient life span. Mutation supply principles also apply to drug design. If multiple independent mutations are required to convey resistance to a single drug, then this will reduce the rate at which effective resistant mutants occur, a concept termed “evolutionary distance” in HIV therapy (Vandamme & Camacho, 2011).

For some antibiotics, for example, third‐generation cephalosporins, resistance evolution by mutation alone can be difficult (Lipsitch, 2001). Here, the availability of new genes on mobile genetic elements (MGEs), such as plasmids, can replace mutation in terms of the critical supply of genetic novelty. Thus, small increases in carriage of resistance MGEs could have profound consequences in terms of providing the essential variation upon which selection can act. For example, while epidemiological modelling of the effect of antibiotic usage in animals on AMR in humans is rather equivocal (Blanquart, 2019), studies that have specifically looked at mutation supply have concluded that antibiotic usage in animals led to the earlier appearance of resistance, precisely when the supply of genetic novelty was limiting (Smith, Harris, Johnson, Silbergeld, & Morris, 2002). This suggests that RM strategies to restrict spontaneous evolution of resistance (by high doses, for instance) can be undermined by rare horizontal gene transfer from large reservoirs of antibiotic‐resistant bacteria in animals or in the environment.

Although horizontal gene transfer can undermine the value of combinations (Bonhoeffer et al., 1997), combinations could still be useful in combatting MGE‐acquired resistance, provided the transfer rates of resistance genes are very low. Since transfer rates are driven by the prevalence of MGEs (Levin, Stewart, & Rice, 1979), we return to Rule 1, pre‐emptive action when resistance is rare is essential (Figure 2; Roush, 1998; Vandamme & Camacho, 2011). Drug resistance in TB shows this clearly: prior exposure to single drug regimens is major risk factor for failure of combinations as this increases the frequency of resistance to vital drugs (Monedero & Caminero, 2010). Reducing mutation supply by reducing pathogen population size, on the other hand, is beneficial. For TB, different drugs have greatest effect in different sub‐populations within the lung: isoniazid best targets actively growing bacteria, pyrazinamide best targets those in an acid environment, and rifampicin rapidly kills bacteria breaking out of dormancy (Mitchison, 1985). This complementary action ensures that population sizes are controlled much more effectively with combinations.

While combinations are powerful, their deployment can be more challenging, because there are more assumptions to be met in comparison with other RM strategies (Roush, 1998; Figure 2). The general multidrug assumption to use of chemistries with different modes of action also applies here, but in addition, simultaneous dosing has particular constraints. First, combined drugs should have similar persistence and efficacy. For example, effective insecticide combinations only became possible because genetic engineering facilitated high expression levels of multiple toxins *in planta* (Huang, Andow, & Buschman, 2011). Conversely, a commonly used anti‐malarial combination of artemisinin and mefloquine breaks this RM assumption and Rule 1: artesunate is lost more rapidly from the body than older anti‐malarials (Adjuik et al., 2004; Nosten et al., 2000), while resistance to mefloquine was high when these combinations were first rolled out. Potentially, both these factors contributed to the rise of partial artemisinin resistance (Boni et al., 2016).

A second complication particularly associated with combinations is how different drugs interact. Drugs can act independently, interact synergistically to increase efficacy or interact antagonistically to reduce efficacy. Synergistic interactions are tempting in terms of improved toxicity (Paul et al., 2014), but are bad for resistance management (Lipsitch & Levin, 1997; MacLean, Hall, Perron, & Buckling, 2010; Pena‐Miller et al., 2013; Raymond, Wright, Crickmore, & Bonsall, 2013). This is because single mutations conferring resistance to one drug tend to cancel out the synergistic effects of the second drug, thereby increasing the fitness benefits conferred (Hegreness, Shoresh, Damian, Hartl, & Kishony, 2008; MacLean et al., 2010; Pena‐Miller et al., 2013). Conversely, antagonistic interactions are protective, since mutations that confer resistance to one toxin provide more limited selective benefits (Yeh, Hegreness, Aiden, & Kishony, 2009). Nevertheless, although many drugs interact in vitro (Ankomah, Johnson, & Levin, 2013; Hegreness et al., 2008; Kim et al., 2014; Pena‐Miller et al., 2013), there is not good evidential support for synergistic interactions persisting in vivo*,* except in highly immuno‐compromised patients (Paul et al., 2014; Tamma et al., 2012). This suggests that synergism may not necessarily be a real barrier to deployment of combinations in RM (Fish, Piscitelli, & Danziger, 1995).

Assessing the broader resistance management consequences of combinations is challenging because of discrepancies between in vitro and in vivo work or between clinical studies. For instance, combinations can select for broad‐spectrum resistance in *P. aeruginosa *in vitro (Vestergaard et al., 2016), while a clinical meta‐analysis has shown that combinations reduce the mortality of individuals infected with this bacterium, but not other Gram‐negative species (Safdar, Handelsman, & Maki, 2004). Pharmacodynamic experiments have demonstrated that combinations can limit evolution of resistance (Thomas et al., 1998). Unfortunately, too few clinical trials, or large reviews, have investigated effects of combinations on evolution of resistance in addition to clinical outcomes (Paul et al., 2014; Tamma et al., 2012). A Cochrane review of sepsis treatment concluded that the combinations do not improve patient mortality and that the side‐effects of aminoglycosides in combinations outweigh any clinical benefits (Paul et al., 2014). In contrast, a review of 171 clinical trials, which specifically considered resistance, found that combinations (typically of β‐lactams and aminoglycosides) reduced the emergence of resistance from 5.6% to 3.1% of infections, without adverse clinical outcomes (Fish et al., 1995). Benefits in relation to aminoglycoside‐ or penicillin‐based monotherapies, which led to resistance emerging at least 8% infections, were particularly clear (Fish et al., 1995). To conclude, the RM benefits of combinations are not obvious from routine clinical outcomes (Paul et al., 2014; Safdar et al., 2004), or often from single trials (Fish et al., 1995), and more synthesis of clinical data is needed. Nevertheless, if narrow‐spectrum drugs become available, and if their use can be restricted to humans, combinations are likely to be an effective way of preserving efficacy.

## 5 RULE 4. LOW DOSES DON'T WORK, SHORT COURSES MIGHT

Higher doses of toxins or of antibiotics can impose more intense selection pressure on mutations that confer resistance (Costelloe et al., 2010; Day & Read, 2016; Kouyos et al., 2014). It follows that using the lowest dose possible to achieve a treatment effect could slow the spread resistance (Blanquart, 2019; Kouyos et al., 2014). However, there is danger that reducing doses will have adverse effects on mutation supply (Bell & MacLean, 2018). Resistance mutations confer an advantage over a particular range of doses (Harmand, Gallet, Jabbour‐Zahab, Martin, & Lenormand, 2016; MacLean et al., 2010; Negri, Morosini, Loza, & Baquero, 1994). Importantly, there is a very much greater pool of mutations that can confer resistance to low doses, the principle behind the use of “mutation prevention concentrations” (MPC) in antibiotic therapy (Drlica & Zhao, 2007; Epstein, Gums, & Drlica, 2004; Harmand et al., 2016; Zhou et al., 2000).

While some of the theoretical basis of the MPC has been criticized, for example, the assumption that selection only takes place above minimal inhibitory concentrations (Day et al., 2015), one key prediction is robust: low doses increase mutation supply (Rule 3). Some researchers make a distinction between “on‐target” mutations, that is, that affect the known binding site of a toxic molecule, and “off‐target” mutations that can occur widely throughout the genome (Epstein et al., 2004). There is widespread evidence that low doses select for off‐target mutations (Baquero, Negri, Morosini, & Bla'zquez, 1997; Drlica, 2003; Dubovskiy et al., 2016; Gressel, 2011; Olofsson & Cars, 2007; Pena‐Miller et al., 2013), while low doses of anti‐malarials and pesticides can select for polygenic resistance mechanisms that produce a small shift in dose responses (Barnes, Watkins, & White, 2008; Gressel, 2011).

Biological details can be important here. For instance, plasmid‐borne genes can provide fitness advantages at very low and at very high doses (Alexander et al., 2008; Bottery, Wood, & Brockhurst, 2016; Medaney, Dimitriu, Ellis, & Raymond, 2015; Ojala, Laitalainen, & Jalasvuori, 2013) so that altering antibiotic doses can have limited impact on selection. In management of TB, pharmacogenetic factors warn against low doses. Genetic variation in the speed at which humans break down the key anti‐mycobacterial isoniazid (Pasipanodya, Srivastava, & Gumbo, 2012) means that fast acetylators of this drug are more likely to acquire isoniazid (and multidrug) resistant infections under a low‐dose regime, while high doses equalize this risk across genotypes (Pasipanodya et al., 2012). Pharmacokinetic modelling of the anti‐malarial mefloquine indicates that early use of a low‐dose strategy accelerated the evolution of resistance (Barnes et al., 2008; Simpson et al., 2000). In the case of fluoroquinolone antibiotics, a “stepping stone” model of antibiotic resistance is well established, as mutations selected at low doses can facilitate the acquisition of additional mutations that confer greater levels of resistance (Bell & MacLean, 2018; Epstein et al., 2004).

It is important to make a distinction between treatment dose and treatment duration. Shorter treatment duration can provide many of the benefits of low‐dose regimes such as reduced side‐effects, reduced selection on commensals and environmental bacteria (Day & Read, 2016), without the negative consequences on mutation supply. Theoretically, pulsed doses of antibiotics can also provide benefits for RM, by allowing fitness costs to drive down frequency of resistant bacteria during treatment (Baker, Ferrari, & Shea, 2018). There is increasing evidence that shorter courses with more effective delivery (e.g., inhaled tobramycin) are a better option for control of *P. aeruginosa* in the lungs of cystic fibrosis patients (Waters & Ratjen, 2014). Shorter courses can help prevent selection for resistance to fluoroquinolones (Rees et al., 2015), while low doses and long treatment may be particularly risky (Guillemot et al., 1998; Lin et al., 2014).

Box 2 **Reactive resistance management**

1 Active interventions to reduce the prevalence of resistance after it has arisen could be important complementary RM options. Reducing carriage and transmission of multi‐resistant Gram‐positive bacteria in hospitals have proven value (Derde et al., 2014; Huang et al., 2016), although these have not helped stay the increases in Gram‐negative‐resistant bacteria. Other solutions are still experimental (Table 1). Vaccination may be a promising avenue of research, if it can reduce colonization by resistant bacteria (Baquero, Lanza, Cantón, & Coque, 2014). Biotechnological and bacteriophage‐based approaches can, in principle, specifically target resistance genes or resistance plasmids. Plasmid‐dependent bacteriophage, for instance, can remove lineages carrying resistance plasmids from liquid culture (Ojala et al., 2013; Table 1). Nevertheless, available phage are not effective against the most important plasmids and these phages may not work well in vivo (Mikonranta, Buckling, Jalasvuori, & Raymond, 2019)*.* It is possible to engineer bacteriophage to selectively kill cells carrying particular resistance genes (Bikard et al., 2014). The main constraints here are that bacteriophage has a narrow host range and rapidly selects for resistance. Diverse phage cocktails can extend range and pre‐empt resistance (Forti et al., 2018), as can combinations of phage and antibiotics (Zhang & Buckling, 2012). However, a conventional phage cocktail effectively targeting a species is likely to be far more attractive to regulators and manufacturers than a genetically modified phage cocktail targeting resistant genotypes only.

Plasmids can also be engineered into “displacement vectors,” GM tools that can purge populations of plasmids carrying clinically important resistance genes. This engineering involves de‐activation of part of the toxin–antitoxin “addiction systems” that ensure stable plasmid inheritance and deletion of any pre‐existing resistance genes (Hale, Lazos, Haines, & Thomas, 2010). Nevertheless, experiments in a mouse model show that natural conjugation is insufficient to displace targeted plasmids. Displacement requires the use of an antibiotic driver, which selects for a rare resistance gene encoded on the displacement vector (Kamruzzaman, Shoma, Thomas, Partridge, & Iredell, 2017). Without toxin–antitoxin systems, these unstable vectors are lost rapidly, eventually producing hosts without target plasmid or vector (Kamruzzaman et al., 2017). Despite the drawback of needing an antibiotic driver, this technology could potentially remove key traits from individuals when resistance is a barrier to surgery or chemotherapy, for instance. In general a common limitation of all biotechnological approaches is their reliance on a plasmid or bacteriophage vehicles to spread a genetically modified (GM) tool between resistant bacteria (Table 1).

**Table 1:** Options for reactive resistance management aimed at reducing the prevalence or transmission of AMR in bacterial populations

Strategy	Selective action	Vehicle	Driver	Evidential support
Infection prevention and preventative treatment (Gram‐positive MDR microbes)	Screening of resistant lineages; topical treatment	Antibacterials; disinfectants; infection control	None required	Clinical data
Plasmid displacement	Toxin–antitoxin‐based removal of plasmid groups	Engineered plasmid (“displacement vectors”)	Conjugation or antibiotic selection	In vitro in vitro in vivo
Conjugation‐dependent phage	Pilus‐dependent infection of plasmid carrying cells	Naturally occurring bacteriophage (PRD1)	Lytic infection	In vitro
Selective mortality using phagemids	*CRISPR‐Cas9* targeting of resistance genes	Engineered bacteriophage (“phagemids”)	Lytic infection	In vitro

Note

*Selective action* describes the basis on which antibiotic‐resistant lineages are targeted in preference to susceptible bacteria. *Vehicle* refers to the biological or pharmaceutical agent used to impose mortality or displacement. *Driver* refers to the mechanism used to ensure spread of vehicle within a bacterial population.

## 6 RULE 5. INFORMATION IS POWER

There has been intensive debate on which RM interventions are best, particularly in regard to the mixing and cycling of antibiotics (Beardmore et al., 2017). There is wide theoretical support for strategies that deliver the greatest heterogeneity in drug/toxin exposure, and mixing typically delivers this more effectively (Bergstrom, Lo, & Lipsitch, 2004; Bonhoeffer et al., 1997; REX‐Consortium 2013; Rimbaud, Papaïx, Barrett, Burdon, & Thrall, 2018). However, this has been challenged by recent theory. For example, if RM is delivered naively, with little regard to prevalence of resistance, then cycling or mixing can make things worse by using antibiotics that are largely ineffective because of high levels of resistance (Beardmore et al., 2017). Moreover, the relative benefits of mixing and cycling can depend upon particular parameter values, and these values are likely to be unknown (Beardmore et al., 2017). Given the complexity of the problem, it is also hard to give recommendations on the details of how to deploy RM, for example, in terms of lengths of cycling periods. A key conclusion, however, is that responsive RM based on recent drug susceptibility data can shape more effective stewardship (Beardmore et al., 2017; Takesue et al., 2010, 2006). Given the importance of data, a pragmatic recommendation is to base cycling periods on the periodicity of data availability and on the constraints of human behaviour. For example, while 3‐month periods have proved practicable (Takesue et al., 2010), changing antibiotic regimens too frequently may create problems with implementation.

There are other valuable rules of thumb for multidrug RM. First, a high prevalence of multidrug resistance means these RM strategies are unlikely to work (Hedrick et al., 2008), potentially limiting interventions to transmission management, rapid treatment and reactive RM. Second, the efficacy of different drug regimens should be broadly similar at the start of deployment of RM, and this will avoid ineffective treatments and acceleration of the evolution of resistance (Beardmore et al., 2017). Third, it is hard to know whether cycling strategies will be effective in advance, as these depend on unknowns such as in vivo fitness costs. However, pragmatically, if resistance is not going down during periods of antibiotic withdrawal, then this strategy is not working and something else should be tried. Unlike combinations, which can select for multidrug resistance and impose side‐effects, cycling is a relatively low risk management gambit.

The advent of cheap and powerful sequencing methods and the generation of “big data” can also present opportunities for RM. Nanopore sequencing technology can provide data very rapidly and cut the time required to identify and profile the resistance of isolated microbes, relative to culture‐based approaches (Schmidt et al., 2016; Tamma et al., 2019). There are clear benefits to rapid resistance profiling for patient treatment, although this method cannot effectively characterize single nucleotide resistance mutations or yet reliably distinguish some important allelic variants. From an RM perspective, there are benefits from more rapid treatment of problematic infections, as this limits transmission (Beardmore et al., 2017; Box 1). Rapid resistance profiling could also expand the tool kit of antibiotics available to prescribers and therefore reduce selection pressure on resistance to key drugs (Whittles et al., 2017). Additionally, profound impacts on resistance could also result from the simpler task of species identification, provided this can facilitate prescription of narrow‐spectrum antibiotics.

The integration of infection and resistance prevalence data with mathematically modelling also provides a powerful basis for developing resistance management plans for individual microbes (Whittles et al., 2017). Earlier theoretical work on the transmission and epidemiology of resistance has been instructive in terms of conceptual advances, but has a poorer track‐record in terms of matching theoretical predictions to data (Blanquart, 2019). Exploring RM strategies with well‐parameterized models and collecting key microbiological data on resistance during clinical trials could help shape much more powerful interventions.

## 7 CONCLUSIONS

The significance of the existing prevalence of resistance for management outcomes cannot be overestimated. There are limited proven reactive resistance managment strategies for reducing resistance levels after they have become high (Box 2). High frequencies of resistance mean that intensive care presents an extremely challenging environment for RM, although this has been a favoured area for clinical trials. Counter‐intuitively, primary care may be a much more rewarding context for RM, precisely because resistance frequencies are lower. Here, resistance can be seen as less problematic (van Hecke, Wang, Lee, Roberts, & Butler, 2017), although in some countries primary care accounts for 75% of antibiotic prescribing (PHE, 2016). Most importantly, levels of resistance in the population at large may determine resistance levels on admission to more critical care contexts.

An additional fundamental take‐home message is that it is very difficult to protect a new mode of action if it is deployed singly, that is, unprotected and outside a multi‐tactic RM strategy. Instead of broad resistance management at a national level, we need detailed integrated management plans for every problematic bacterial species, which could organize the protection of any novel or last resort treatments (Whittles et al., 2017). Future management plans need to be supported by regulators with the will to find ways of efficiently licensing more diverse treatment regimens, such as bacteriophage. Finally, it is worth bearing in mind that no drug or management intervention has yet been shown to be resistance‐proof. Confidence in the durability of novel drugs can be poorly supported (Bell & MacLean, 2018; Smith et al., 2018), while some humility in the face of natural selection can ensure that human creativity keeps pace with evolutionary innovation.

## CONFLICT OF INTEREST

None declared.

## DATA AVAILABILITY

There are no data associated with this manuscript.
